# Materia Medica and Therapeutics

**Published:** 1850-10

**Authors:** 


*Materia Medica and Therapeutics.* By Thomas D. Mitchell, A. M., M. D., Professor of the Theory and Practice of Medicine in the Philadelphia College of Medicine, &c. &c. &c. Philadelphia: Lippincott & Grambo, 1850.

This book is evidently the work of a man of considerable experience, and professes to contain the substance of the author's lectures on Materia Medica and Therapeutics, as delivered in the Medical Department of Transylvania University, in eleven successive winters. As an enlarged and philosophical treatise on Therapeutics, we must say, that it does not come up to our ideas on the subject. The alphabetical arrangement which the author has chosen in preference to a classification of remedies having an analogous Therapeutic application, necessarily breaks the subject too much into fragments, and gives us merely a dry detail of the remedial effects of individual medicinal agents, which, though excellent in themselves, showing great research on the part of the author, and highly useful to the student, does not in fact teach any thing more than would a well arranged Dispensatory.

As a work on Therapeutics, in the extended and scientific sense of the word, we therefore do not approve of Dr. Mitchell's book; as an abridged Dispensatory, with practical Therapeutic suggestions, we do approve of it highly, and think that the research and labor which must have been devoted to the compilation and arrangement of such a mass of material, as honorable to the author as it is creditable to the profession of which he is a worthy member. The great fault of the book, in our estimation, is, a certain want of dignity and good taste in the general tone of the details; a disposition on the part of the author to raise a monument to his own merits at the expense of the profession; an assumption of superior sagacity and intelligence, the existence of which Dr. M. may in the course of his professional career have often *felt*, but the expression of which, we think, might have been alloyed with advantage by "some few cold drops of modesty." These remarks we make in no ill-natured spirit, but merely to express regret that a man of talent should mar a good work by vanity. For, though the foible is displeasing, we can say of the author as Dr. Johnson said of David Garrick, whom some of his cotemporaries were ridiculing for his conceit: "yes, gentlemen, David is vain, but he has the advantage of most of us in having a great deal to be vain of."

The book contains 730 pages, of as varied material as can well be imagined; therefore we must of necessity content ourselves and our readers with a few quotations illustrative of the author's manner of treating his subject. The Toxicological part of the work is excellent; the methods recommended for the detection of poisons; the preparation and application of antidotes, and the general treatment of patients suffering under their influence, are all good; so much so, that we think that this part of the book alone, whatever its other merits may be, entitle it to high praise, and to a space on the shelf of every medical library.

The alphabetical arrangement, though in our opinion disadvantageous to a strictly Therapeutical work, in which, as we before observed, the massing together of analogous remedies, saves the necessity of frequent and tedious repetition, and gives, we think, a wider and bolder view of the subject, is well adapted to the Materia Medica and Toxicological part of the work, and has enabled the author to give the minute detail of an amount of material which otherwise would have been impossible, without making it too voluminous; which, as we presume the author intended it as a text book for the students of his class, would not have answered his purpose.

The details of Dr. Mitchell's personal experience in the use of remedies are generally good, practical, sensible and reliable, but we object to his constant quotations from Braithwaite's Retrospect, Ranking, and the like not that we do not esteem these works as they deserve; on the contrary we think that they sometimes contain much useful information; but they likewise abound in much mere rubbish; new remedies old as the eternal hills, that have been resurrectionized; again buried, and again called into life, to astonish doctors with large organs of wonder and credulity, and to torment their patients.

The fact is, the majority of the contributors to such publications are men of some talent, struggling for a practice; they cannot advertise in the newspapers, because that would be infra dig., and they would by such a proceeding lose caste, therefore the medical journals are the only orthodox means by which they can make themselves known to the public; thus they are constantly on the alert to drag their "soi disant" new modes of treatment before the public, "with twenty mortal murders on their heads to push us from our stools," and which, like Shakespeare's cauldron, manufactured phantoms—

> "Show his eyes and grieve his heart,
> Come like shadows, so depart."

And all this is good and useful in its way; but an over and credulous estimation of such reports is worse than useless, and we contend that it is an over estimation to quote them in standard works, unless proved by the touchstone of experience, as high and reliable authority.

The following quotation is from the author's introduction:

"Possibly some of my readers may be disposed to say that this book should have been called a dispensatory. But having the christening in my own hands, it has seemed good to me to give it the title it bears, and as I think most appropriately. If a dispensatory be not a work on materia medica, why is it made a text book by professors, who teach this department of medical science? The fact is too palpable to be misunderstood by men of sound common sense, that a good dispensatory cannot be a bad exhibit of materia medica; and it is equally manifest that a well digested work on materia medica and therapeutics, even though alphabetically arranged, cannot be a serious hinderance to the study of the teachings of a dispensatory. Vastly fond am I of judicious and real distinctions, but I confess that I have no special regard for a distinction that hardly implies a difference."

We have already given our reasons for considering the alphabetical arrangement as disadvantageous to an enlarged view of the science of therapeutics, and therefore shall make no further comment.

The subjoined remarks upon the subject of the antagonism of poison and disease, are well and philosophically written. Every medical man of experience must be aware of their truth.

"The point to which I desire to call attention specifically, is one that has entered into my public teachings for many years, and I am free to confess that I do not yet comprehend it fully. I refer to the fact about which there can be no difference of opinion (I mean as to its existence) that decidedly poisonous doses, so far as bulk or weight are concerned, have been frequently swallowed without material injury, and that, too, independently of any condition of the stomach sufficient to account for the result. The doctrine that has appeared to me as the true solution of the problem, is that in the most striking cases on record, the otherwise poisonous dose has spent its force on the morbid action present in the system, whatever that may have been, and in this way its legitimate character has not been developed. I have not met with a single direct reference to this view of the case anywhere, excepting in a short article lately published by Dr. Beck, in which there was an incidental allusion to it. I am aware of the effects of habit in controlling the mischievous action of poisons so as to render them quite harmless; but I have no reference to this agency on the present occasion, for that could not meet the difficulty."

"The case most familiar to the profession, illustrative of this doctrine, is the administration of large doses of tartar emetic in the treatment of pneumonic inflammation. Here the dose is often so great as to be exceedingly hazardous if its operation were restricted to the stomach. The idea of tolerance is associated necessarily with the fact that some other organ besides the stomach is to participate in the agency of the remedy; that other organ is the hinge which feels the influence of the medicine in its restoration to the condition of health. Now although we cannot demonstrate all this just as if it were a problem in Euclid, we are compelled to believe that the salutary influence of great doses of tartar emetic in this disease involves the principles which it is our purpose to illustrate.

We do not desire to be understood here as advocating this plan of curing pneumonia, but simply as attempting to account for the result by the obvious antagonism of disease and poison."

The mammoth doses of nitrate of potassa administered latterly in cases of acute rheumatism, are given by Dr. Mitchell as another illustration of this point; and he might have added the enormous doses of calomel which have been administered in fevers, and in Asiatic cholera, without producing salivation.

In speaking of tobacco as an antidote, or a remedy in poisoning by arsenic, he writes as follows:

"A portion of arsenic fully sufficient to kill under ordinary circumstances, was counteracted by the use of tobacco and without any emetic action. What were the facts with regard to the pathological condition of the stomach? The arsenic had commmenced its appropriate action beyond doubt, as the symptoms evinced: there was very probably set up true inflammation, such as arsenic is competent to establish. How then were the patients saved? The tobacco was given in strong infusion, expressly with the design of vomiting, and so dislodging the poison. But the emetic action failed entirely, and yet the patients were restored after comparatively little suffering.

I know that it has been said that the arsenic and tobacco formed a neutral mixture, in which the poisonous property of both articles was lost or nullified. But it is more probable that the intrinsic character of tobacco failed to display itself, because its power was spent on the existing morbid condition of the stomach and bowels."

The author, in treating of counter irritation with tartar emetic, recommends the following combination:

| | | |
|---|---|---|
| ℞. | Emet. tart. | ℈i. |
| | Oil Olivar. | ℥ss. |
| | Oil Croton. | ʒss. Mix. |

We must confess that we look upon this prescription as rather heroic. Croton oil, even well diluted, has frequently in our experience, produced very severe effects; emetic tartar, as every body knows, will often produce most painful ulcerations, therefore we do not understand why they should be combined. Dr. Mitchell is evidently a good chemist, and may have discovered, or fancy that he has discovered (which is the same thing now a days,) that croton oil and tartar emetic exert a modifying influence on each other when in combination; but as he has not favored us with any such developement, we ourselves shall continue to use them separately. We must also object, by the way, to the exceedingly unscientific jumble of languages in this formula, for which we know no authority. We are charitable enough to attribute it to oversight on the part of the author, but it is a dangerous example to set before a beginner.

One author has given us a very good article upon the uses and abuses of alcohol, in the course of which he says, and every member of the profession will agree with him, that it is the duty of doctors to discourage drunkenness: but we protest against the bad taste of the following remarks:

"On account of its active solvent power, (alcohol,) the books abound with tinctures, and there has been too great a willingness to exhibit them. But, inasmuch as a large majority of the sots have been made so by the doctors through the agency of alcoholic medicines, it is the duty of the profession to correct the evil as speedily as the nature of the case will allow."

We have heard this charge made in the lecture-room, and always regarded it as puerile and ridiculous: such excuses may have been made by sots, who think that by accusing the doctors, they excuse their own brutal vices. But what man of common sense will ever believe that a few teaspoonfuls of a medicinal tincture, taken occasionally, ever truly made a drunkard? No; the fellow who could have the impudence to make such a charge, would have been a sot under any circumstances. The thief might just as rationally excuse his career of crime, by stating that having been once, by his wicked father or employer, left alone with a cent, he took it, and could never again refrain. A tendency so strong is the disease itself, not developed, perhaps, but it is there. As Lord Byron says of friendship between the two sexes,

"It is love full fledged, waiting for a fine day to fly."

Cod liver oil seems to be a favorite subject with Dr. Mitchell; for though he does not give us much of his own experience in its use, which we regret, we should have more faith in it than in Dr. Williams' flourish of trumpets, quoted at length by our author, which reads to us very much like the rhapsody of a very well meaning and learned gentleman on the back of a favorite hobby-horse, which he has previously determined to ride nobody knows where.

Cod liver oil, it would appear, is the fashion, and, as was for some time the case with the iodide of potassium, is now prescribed for every thing. Upon the credit of various high authorities it will cure the following diseases:—Consumption, Bronchitis, Pleurisy, Neuralgia, Liver Complaint, Rheumatism, Tertiary Syphilis, Scrofula, and all its incidental ailments, Gout, Dyspepsia, Lepra, Impetigo, Porrigo, &c., &c., &c. So that, in short, the fabulous Elixir of the alchymist is at last realized, and in the hands of the speculating apothecaries, who swept the market of all the stinking oil they could find, has doubtless produced a golden harvest.

We have tried the remedial powers of this oil, and found it of service in some scrofulous affections, but we are sorry to say that we have never yet found it of use in any undoubted case of tubercular phthisis. Still, we are willing to continue the trial, and never let pass a favorable opportunity of doing so; for it is our wish to see this remedy, if it be one for genuine phthisis, take its proper place.

But such a system of puff and exaggeration as has been lately carried on in most of the journals upon this subject, is really as pernicious to the interests of humanity, and the true dignity of medical science, as the worst quackery that ever imposed on the credulity of mankind.

When on the subject of calomel, the author makes the following remarks, which are perhaps judicious, but which we fear might be construed into an apology for ignorance on the part of some blundering or uneducated shop boy.

"As a thing of mere expediency, not to say safety, I hold the word calomel to be preferable, in our written prescriptions, to any other term; and the same remark is of equal force touching corrosive sublimate. By these common names all persons, boys or men, engaged in dispensing medicines, know the one and the other. But all do not comprehend the more correct technicals, which are oftener employed to display the imaginary knowledge of the doctor, than because they are really to be preferred. Fewer blunders would occur, if we invariably used the old terms."

We regret that want of space prevents our quoting more extensively from Dr. Mitchell's book. Notwithstanding the fault which we have presumed to find with certain portions of it, we cannot conclude without repeating our good opinion of it as a whole—recommending it heartily to the perusal of all persons interested in such subjects. To the young practitioner and the medical student we think it will prove a valuable assistant.

